# Elevated Serum Alpha-Fetoprotein Is a Significant Prognostic Factor for Patients with Gastric Cancer: Results Based on a Large-Scale Retrospective Study

**DOI:** 10.3389/fonc.2022.901061

**Published:** 2022-06-29

**Authors:** Zhouwei Zhan, Bijuan Chen, Jiami Yu, Jingxian Zheng, Yi Zeng, Mingyao Sun, Li Peng, Zengqing Guo, Xiaojie Wang

**Affiliations:** ^1^Department of Medical Oncology, Fujian Medical University Cancer Hospital, Fujian Cancer Hospital, Fuzhou, China; ^2^Department of Radiotherapy, Fujian Medical University Cancer Hospital, Fujian Cancer Hospital, Fuzhou, China; ^3^Department of Gastrointestinal Surgery, Fujian Medical University Cancer Hospital, Fujian Cancer Hospital, Fuzhou, China; ^4^Department of Clinical Nutrition, Fujian Provincial Hospital, Fuzhou, China; ^5^Department of Diagnostic Radiology, Fujian Medical University Cancer Hospital, Fujian Cancer Hospital, Fuzhou, China

**Keywords:** gastric cancer, alpha-fetoprotein (AFP), clinicopathological features, liver metastasis, prognosis

## Abstract

**Objectives:**

The aim of this work is to study the clinicopathological features and prognostic factors of serum alpha-fetoprotein (AFP)–positive gastric cancer (GC).

**Methods:**

A cohort study including 2,318 patients with GC who underwent radical surgery from January 2008 to December 2015 was retrospectively analyzed. Patients were divided into two groups according to preoperative serum AFP values: 191 patients with AFP-positive GC (AFP > 20 ng/ml, 8.24%) and 2,127 patients with AFP-negative GC (AFP ≤ 20 ng/ml, 91.76%). The clinicopathological features and prognostic factors were explored.

**Results:**

Compared with AFP-negative GC, AFP-positive GC had higher rates of liver metastasis, lymph node metastasis, venous invasion, and nerve invasion (all *P* < 0.05). The 5-year OS, DFS, and mLMFS of AFP-positive GC were shorter than AFP-negative GC (55.00% vs. 45.04%, *P* < 0.001; 39.79% vs. 34.03%, *P* < 0.001; 13.80 months vs. 16.25 months, *P* = 0.002). In whole cohort, multivariate analysis found that serum AFP levels (positive vs. negative), pT stage, pN stage, nerve invasion (yes or no), and venous invasion (yes or no) were independent prognostic factors. Serum AFP levels (20–300 ng/ml vs. 300–1,000 ng/ml vs. >1,000 ng/ml), pT stage, pN stage, and venous invasion (yes or no) were independent prognostic factors in AFP-positive GC.

**Conclusion:**

Liver metastases and venous invasion are more likely to occur in AFP-positive GC and lead to poor prognosis. Serum AFP level is an independent prognostic factor in patients with GC. As the level of AFP increases, the prognosis becomes worse.

## Introduction

Alpha-fetoprotein (AFP) is a specific tumor marker and commonly used in the diagnosis of hepatocellular carcinoma and yolk sac tumor (1, 2). Other tumors, such as gastric cancer (GC), colorectal cancer, lung cancer, and ovarian cancer, can also lead to elevated AFP (3). The most common is GC (3). AFP-positive GC, which should be differentiated from other ordinary-type of GC, is a relatively rare type of gastric malignancy and was initially reported by Bourreille et al. in 1970 (4). Compared with ordinary-type of GC, AFP-positive GC is more aggressive, and more prone to early lymph node metastasis and distant metastasis. In particular, the incidence of liver metastasis is much increased, and the prognosis is worse (5). In recent years, the treatment of AFP-positive GC has been gradually emphasized. However, studies on the clinicopathological characteristics of AFP-positive GC and the prognostic factors are rare. This study is intended to compare the clinical-pathological characteristics and prognosis of patients with AFP-positive GC and AFP-negative GC and to explore the risk factors that affect the prognosis of patients with AFP-positive GC.

## Materials and Methods

### Study Design and Patients

From January 2008 to December 2015, 2,318 patients with histologically confirmed primary GC who underwent R0 resection at Fujian Medical University Cancer Hospital, Fujian Cancer Hospital, were retrospectively enrolled in the study. All patients had complete clinical pathology data and received complete follow-up data. Patients were excluded if they underwent any neoadjuvant therapy before the operation or R1/R2 resection or had any other AFP-producing conditions such as active or chronic hepatitis, liver cirrhosis, and hepatocellular carcinoma or with metastatic disease. Serum AFP values were measured preoperatively using a Roche electrochemiluminescence instrument cobas e 602. The cutoff value for serum AFP was 20 ng/ml. Serum AFP > 20 ng/ml is defined as AFP-positive GC; serum AFP ≤ 20 ng/ml is defined as AFP-negative GC. The study protocol following the ethical guidelines of the 1995 Declaration of Helsinki was approved by the ethics committee of Fujian Medical University Cancer Hospital, Fujian Cancer Hospital (ethical approval number K2021-100-01).

### Baseline and Follow-Up Assessment

The following parameters were examined: age, sex, serum AFP, CEA, CA19-9, CA72-4 level, type of operation, tumor location, tumor size, pathological differentiation, TNM classification, vessel invasion, nerve invasion, Ki-67, and postoperative chemotherapy. Tumor staging was done according to the TNM classification of the AJCC (seventh edition) (6, 7). The follow-up program schedule for all patients comprised a regular physical examination and laboratory blood tests including tumor markers, chest CT, abdominal pelvic CT, or MRI examination (every 3 months in the first postoperative year, every 6 months in the second post-operative year, and annually thereafter for at least 5 years).

The follow-up period was calculated from the day of surgery to the last follow-up date. The deadline for follow-up is October 1, 2020. Overall survival (OS) was defined as the time from surgery to death and otherwise the patients were censored. Recurrence-free survival (RFS) was identified as the time from surgery to recurrence evaluation. Liver metastasis-free survival (LMFS) was described as the time from surgery to liver metastasis occurring. OS, RFS, and LMFS were retrospectively calculated and analyzed according to patients’ records.

### Statistical Analysis

All data were statistically analyzed using SPSS 23.0 (SPSS Inc., Chicago, IL, USA). Graphs were created by GraphPad Prism v.9.0.0 (La Jolla, CA, USA). X-tile 3.6.1 software (Yale University, New Haven, CT, USA) (8) was used to determine the optimal cut-off values for age, tumor size, Ki-67 expression, and serum AFP values in GC cohort. A chi-square test was used for comparison of clinicopathological factors between AFP-positive GC and AFP-negative GC. Cumulative survival rates were calculated according to the Kaplan–Meier method. Log-rank test was used for univariate survival analysis, and Cox regression model was used for multivariate survival analysis. A two-tailed *P* asset value <0.05 was considered statistically significant. Multivariate analyses were performed to figure out the independent prognostic factors using the Cox proportional hazards mode by backward elimination of insignificant variables. Host factors including age (>60 yes or no) and gender (male or female) acted as covariates. The pT stage, pN stage, venous invasion (yes or no), nerve invasion (yes or no), Ki-67–positive rate (>50% yes or no), tumor size (>3cm yes or no), pathological differentiation (highly-moderately or poorly differentiation), and Lauren’s classification (intestinal or diffuse-mixed) were used as covariates when analyzing the serum AFP variable (20–300 vs. 300–1,000 vs. >1,000). Significance analysis adopted log-rank test. A two-tailed *P* asset value <0.05 was considered statistically significant.

## Results

### Patients’ Characteristics of AFP-Positive GC and AFP-Negative GC

A total of 2,318 patients with GC were enrolled. According to the X-tile plots and previous literature reports (5, 9), the optimal cutoff point for serum AFP values was 20 ng/ml. There were 191 AFP-positive GC (AFP > 20 ng/ml, 8.24%) and 2,127 AFP-negative GC (AFP ≤ 20 ng/ml, 91.76%) before surgery. The difference of incidence of liver metastasis, lymph node metastasis, venous invasion, and nerve invasion between the two groups was statistically significant (*P* < 0.05, **Table 1**). More patients with AFP-positive GC received adjuvant chemotherapy after surgery (70.2% vs. 63.3%, *P* = 0.004). There were no significant differences between the two groups in terms of gender, age (year), ECOG-PS score, tumor site, tumor size, gastrectomy, pathological differentiation, pT stage, pTNM stage, lauren type, elevated CEA, CA19-9, CA72-4 proportion, and Ki-67–positive rate (*P* > 0.05, **Table 1**).

**Table 1:** Comparison of clinicopathologic characteristics of patients with AFP-positive GC and AFP-negative GC.

Characteristic		AFP-positive (n = 191, %)	AFP-negative (n = 2,127, %)	*χ^2^*	*P*-value
Sex	Male	125 (65.4%)	1,262 (59.3%)	2.725	0.099
	Female	66 (34.6%)	865 (40.7%)		
Age (year)	<60	87 (45.5%)	989 (46.5%)	0.063	0.801
	>60	104 (54.5%)	1,138 (53.5%)		
ECOG-PS	0	60 (31.4%)	718 (33.8%)	1.139	0.566
	1	78 (40.8%)	786 (37.0%)		
	2	53 (27.7%)	623 (29.3%)		
Tumor site	Esophagogastric junction	43 (22.5%)	599 (28.2%)	4.160	0.245
	Gastric body/fundus	48 (25.1%)	437 (20.5%)		
	Gastric antrum	58 (30.4%)	665 (31.3%)		
	Leather stomach	42 (22.0%)	426 (20.0%)		
Gastrectomy	Subtotal	73 (38.2%)	868 (40.8%)	0.487	0.485
	Total	118 (61.8%)	1,259 (59.2%)		
Tumor size (cm)	<3	100 (52.4%)	1,053 (49.5%)	0.681	0.409
	>=3	91 (47.6%)	1,074 (50.5%)		
CEA (ng/ml)	≤5	72 (40.8%)	887 (41.7%)	1.159	0.282
	>5	119 (59.2%)	1,240 (58.3%)		
CA19-9 (U/ml)	≤30	77 (40.3%)	894 (42.0%)	0.212	0.645
	>30	114 (59.7%)	1,233 (58.0%)		
CA72-4 (U/ml)	≤6.9	86 (45.0%)	936 (44.0%)	0.074	0.786
	>6.9	105 (55.0%)	1,191 (56.0%)		
Pathological differentiation	Highly- Moderately	90 (47.1%)	953 (44.8%)	0.380	0.538
	Poorly	101 (52.9%)	1,174 (55.2%)		
pT stage	T1	33 (17.3%)	317 (14.9%)	3.160	0.368
	T2	31 (16.2%)	325 (15.3%)		
	T3	47 (24.6%)	455 (21.4%)		
	T4	80 (41.9%)	1,030 (48.4%)		
pN stage	N0	44 (23.0%)	680 (32.0%)	6.511	0.010
	N+	147 (77.0%)	1,447 (68.0%)		
pTNM stage	I	31 (16.2%)	349 (16.4%)	0.435	0.804
	II	65 (34.0%)	770 (36.2%)		
	III	95 (49.7%)	1,008 (47.4%)		
Lauren type	Intestinal	87 (45.6%)	953 (44.8%)	0.039	0.843
	Diffuse-Mixed	104 (54.4%)	1,174 (55.2%)		
Venous invasion	Yes	101 (52.9%)	888 (41.7%)	8.876	0.003
	No	90 (47.1%)	1,239 (58.3%)		
Nerve invasion	Yes	138 (72.3%)	1,293 (60.8%)	9.746	0.002
	No	53 (27.7%)	834 (39.2%)		
Ki-67	<50%	102 (53.4%)	1,151 (54.1%)	0.036	0.850
	≥50%	89 (46.6%)	976 (45.9%)		
Adjuvant chemotherapy	Yes	134 (70.2%)	1,268 (63.3%)	8.150	0.004
	No	57 (29.8%)	859 (36.7%)		
Liver metastasis	Yes	89 (46.6%)	530 (24.9%)	42.084	0.000
	No	102 (53.4%)	1,597 (75.1%)		

### Univariate and Multivariate Survival Analyses in GC

The median follow-up of the whole group was 61 (1–102) months. Univariate analyses revealed that pT stage, pN stage, venous invasion (yes or no), nerve invasion (yes or no), Ki-67–positive rate (>50% yes or no), tumor size (>3cm yes or no), Lauren type (intestinal or diffuse-mixed), and serum AFP levels (positive vs. negative) were prognostic factors for GC (*P* < 0.05, **Table 2**). Multivariate analyses using stepwise Cox regression procedures revealed that serum AFP levels (positive vs. negative), pT stage, pN stage, nerve invasion (yes or no), and venous invasion (yes or no) were independent prognostic factors (*P* < 0.05, **Table 2**).

**Table 2:** Univariate and multivariate survival analyses in 2,318 patients with GC.

Factor	Univariate *P-*value	Multivariate		
		HR	95%CI	*P*-value
Sex (Male vs. female)	0.146			
Age (≤60 vs. >60 year)	0.313			
ECOG-PS (Score 0 vs. 1 vs. 2)	0.133			
Tumor site (esophagogastric junction vs. *g*astric body/fundus vs. gastric antrum vs. leather stomach)	0.080			
Gastrectomy (subtotal vs. total)	0.535			
Tumor size (<3 vs. ≥3 cm)	0.039			
CEA (≤5 vs. >5 ng/ml)	0.462			
CA19-9 (≤30 vs. >30 U/ml)	0.318			
CA72-4 (≤6.9 vs. >6.9 U/ml)	0.266			
Pathological differentiation (highly*-*moderately vs. poorly)	0.651			
pT stage (T1 vs. T2 vs. T3 vs. T4)	0.000	1.270	1.172–1.376	0.000
pN stage (N0 vs. N1 vs. N2)	0.000	2.196	2.043–2.361	0.000
Lauren type (intestinal vs. diffuse-mixed)	0.048			
Venous invasion (yes vs. no)	0.035	1.171	1.027–1.334	0.018
Nerve invasion (yes vs. no)	0.000	0.858	0.755–0.975	0.019
Ki-67 (<50% vs. ≥50%)	0.000			
Adjuvant chemotherapy (yes vs. no)	0.899			
Serum AFP (positive vs. negative)	0.000	0.604	0.496–0.737	0.000

CI, confidence interval; HR, hazard ratio.

### Prognosis of AFP-Positive GC and AFP-Negative GC

**Figure 1:**
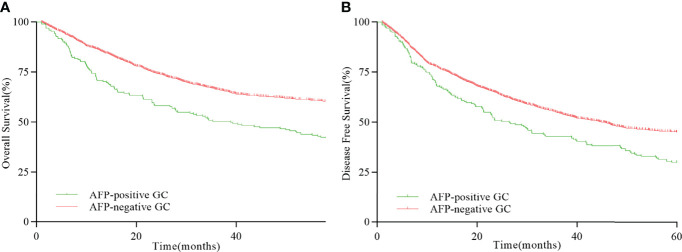
**(A)** Comparison of OS rates between AFP-positive GC and AFP-negative GC (*P* < 0.001). **(B)** Comparison of DFS rates between AFP-positive GC and AFP-negative GC (*P* < 0.001).

**Figure 2:**
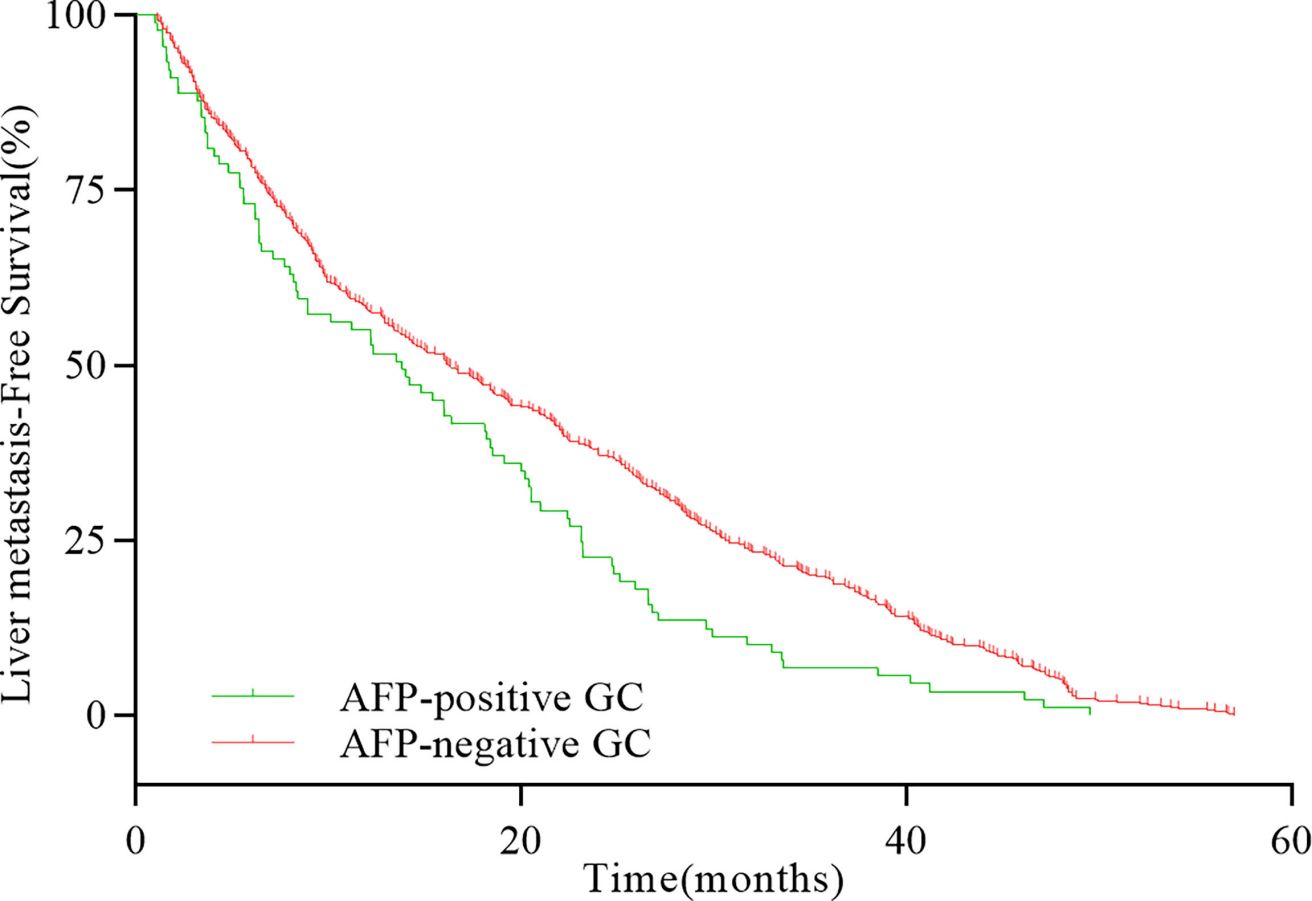
Comparison of LMFS rates between AFP-positive GC and AFP-negative GC with liver metastases (*P* = 0.001).

The 5-year OS and DFS of patients with whole GC were 53.75% and 44.13%, respectively. The 5-year OS and DFS of AFP-positive GC were 39.79% and 34.03%, whereas the 5-year OS and DFS of AFP-negative GC were 55.00% and 45.04%, respectively. The differences of the 5-year OS and DFS between AFP-positive GC and AFP-negative GC were statistically significant (OS, HR = 1.511, *P* < 0.001, **Figure 1A**; DFS, HR = 1.354, *P* < 0.001, **Figure 1B**). In patients with liver metastasis, the median LMFS (mLMFS) was calculated. The mLMFS of AFP-positive GC was much shorter than patients with AFP-negative GC (13.80 months vs. 16.25 months, HR = 1.425, *P* =0.002, **Figure 2**).

### Univariate and Multivariate Survival Analyses in AFP-Positive GC

**Figure 3:**
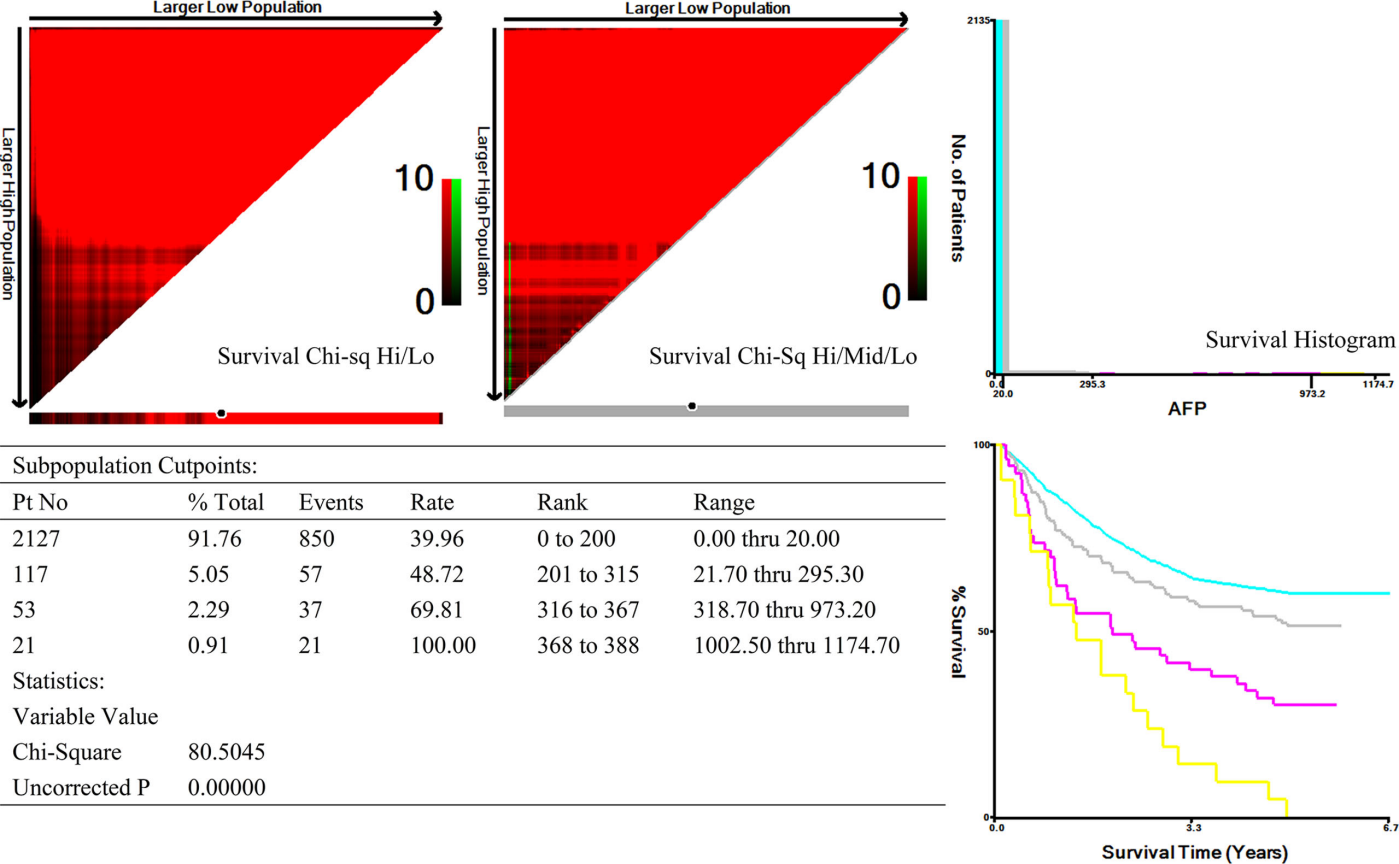
X-tile analysis of overall survival according to serum AFP values.

**Figure 4:**
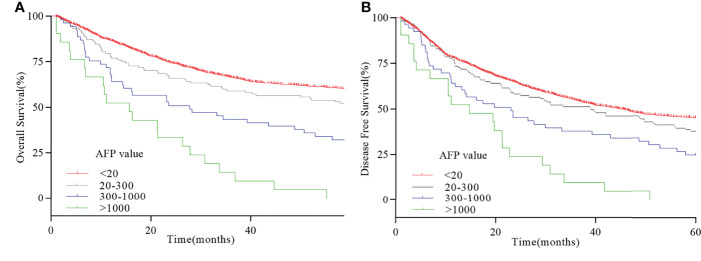
Survival curve of serum AFP-positive GC. **(A)** 5-year OS curve with different serum AFP values. **(B)** 5-year DFS curve with different serum AFP values.

X-tile analysis was performed to identify the optimal cutoff points of serum AFP values for survival analysis. As showed in **Figure 3**, 300 and 1,000 ng/ml were identified as the optimal cutoff values. Univariate analyses revealed that pT stage, pN stage, venous invasion (yes or no), Ki-67–positive rate (>50% yes or no), tumor size (>3cm yes or no), pathological differentiation (highly-moderately or poorly differentiation), Lauren type (intestinal or diffuse-mixed), and serum AFP levels (20–300 ng/ml vs. 300–1,000 ng/ml vs. >1,000 ng/ml) were prognostic factors for AFP-positive GC (*P* < 0.05, **Table 3**). Multivariate analyses using stepwise Cox regression procedures revealed that serum AFP levels (20–300 ng/ml vs. 300–1,000 ng/ml vs. >1,000 ng/ml), pT stage, pN stage, and venous invasion (yes or no) were independent prognostic factors (*P* < 0.05, **Table 3** and **Figure 4**).

**Table 3:** Univariate and multivariate survival analyses in 191 patients with AFP-positive GC.

Factor	Univariate *P-*value	Multivariate		
		HR	95%CI	*P*-value
Sex (male vs. female)	0.816			
Age (≤60 vs. >60 year)	0.082			
ECOG-PS (score 0 vs. 1 vs. 2)	0.944			
Tumor site (esophagogastric junction vs. gastric body/fundus vs. gastric antrum vs. leather stomach)	0.615			
Gastrectomy (subtotal vs. total)	0.232			
Tumor size (<3 vs. ≥3cm)	0.043			
CEA (≤5 vs. >5 ng/ml)	0.311			
CA19-9 (≤30 vs. >30 U/ml)	0.362			
CA72-4 (≤6.9 vs. >6.9 U/ml)	0.440			
Pathological differentiation (highly*-*moderately vs. poorly)	0.031			
pT stage (T1 vs. T2 vs. T3 vs. T4)	0.000	1.414	1.162–1.720	0.000
pN stage (N0 *vs* N+)	0.000	3.158	1.626–6.133	0.001
Lauren type (intestinal vs. diffuse-mixed)	0.001			
Venous invasion (yes vs. no)	0.000	0.525	0.355–0.776	0.001
Nerve invasion (yes vs. no)	0.737			
Ki-67 (<50% vs. ≥50%)	0.014			
Adjuvant chemotherapy (yes vs. no)	0.122			
Serum AFP values (20–300 vs. 300–1,000 vs. >1,000 ng/ml)	0.000	1.554	1.227–1.969	0.000

CI, confidence interval; HR, hazard ratio.

## Discussion

The present retrospective study investigated the serum AFP level as an independent prognostic factor for GC. A high level of serum AFP (>20 ng/ml) was associated with a high incidence of lymph node metastasis, venous and nerve invasion, liver metastasis, and a poor prognosis. Liver metastasis was frequently and earlier observed in patients with AFP-positive GC. Different serum AFP values play as independent prognostic factor in AFP-positive GC. Special attention should therefore be paid to patients with AFP-positive GC. These results are consistent with previous findings that AFP production predicts worse outcomes in patients with GC (9, 10).

The definition of AFP-positive GC varies in different studies. Some studies define it as AFP-positive GC based on the immunohistochemical result during postoperative histological workup (11). Others defined AFP-positive GC depending on preoperative serum AFP levels (5). The incidence of AFP-positive GC is 1.3%~15.0% worldwide (11–13). An analysis from Liu et al. indicated that patients with elevated serum AFP level preoperatively were proved to be AFP-positive GC by immunohistochemistry, the ratio was 93.7%. There was no statistical difference in OS between patients with positive IHC or just serum AFP elevation (13). In our study, AFP-positive GC was defined as the preoperative serum AFP elevation >20 ng/ml (5, 14), and the prevalence was 8.24% in a period of seven years, which was identical to that of other studies.

As a rare type of GC, AFP-positive GC was found to be more aggressive than AFP-negative GC and was characterized by a high rate of metastasis to the liver and lymph nodes (14, 15). AFP-positive GC have a high proliferative activity, weak apoptosis, and rich neovascularization that render them very aggressive with a poor prognosis (16). The 1-, 3-, and 5-year survival rates of AFP-positive GC were 53%, 35%, and 28%, respectively (13). The incidence of lymph node metastasis (11, 17, 18) and venous invasion (11, 19) was higher than AFP-negative GC, which was similar to previous research results. In addition, we also observed that the incidence of nerve invasion is also higher than that of AFP-negative GC. A meta-analysis study has confirmed that nerve invasion was an independent prognostic factor for GC, as well a predictor for recurrence of patients with GC who had undergone curative resection (20). The presence of nerve invasion was closely related to lymphatic vessel invasion and blood vessel invasion. However, nerve invasion is not an independent prognostic factor for AFP-positive GC, whereas vascular invasion is one of the independent prognostic factors in AFP-positive GC. Vascular invasion is a critical step in tumor cell dissemination and metastasis and was reported to be associated with lymph node metastasis, advanced T stage, and poor prognosis. It is now usually accepted as a prognostic factor independent of tumor stage, grade of differentiation, or lymph node involvement (21–24). This is consistent with what we observed in AFP-positive GC. The high probability of vascular invasion in AFP-positive GC may play as an important role in the occurrence of liver metastasis and poor prognosis (25). Compared with AFP-negative GC, the proportion of patients who received adjuvant chemotherapy was higher in AFP-positive GC. However, both univariate regression analysis and multivariate analysis using stepwise Cox regression procedures demonstrated that the acceptance of adjuvant chemotherapy was not an independent prognostic factor.

Liver metastasis was reported to occur in 44.1% to 90.9% of AFP-positive GC (10, 11), which was similar to that in our study (46.6%). Furthermore, in our study, higher level of serum AFP values seemed more likely to be associated with early occurrence of liver metastases during disease progression. Hirajima et al. reported that the prognosis of AFP-positive GC and AFP-negative GC was similar according to the presence or absence of liver metastasis. Higher rate of tumor metastasis in the liver after surgery may result in poor outcomes of AFP-positive GC (11). However, they pointed out that liver metastasis was the only independent prognostic factor in AFP-positive GC and AFP-positivity was not an independent prognostic factor (11). Similar results have been observed in other studies (12, 26). Different methods used for the measurement of serum AFP values, as well as regional or racial differences, may result in this discrepancy. AFP-positive GC was defined by immunohistochemical analyses and only 23 AFP-positive GC was reported in the study of Hirajima et al. (11). At present, most scholars believe that AFP immunohistochemistry is not a necessary qualification for diagnosis of AFP-positive GC. Elevated serum AFP in AFP-positive GC may be derived from specific AFP in the gastrointestinal tract (27, 28). Some patients with AFP-positive GC with poor prognosis may be ignored in the study of Hirajima et al. Furthermore, the sample size of patients with AFP-positive GC included in their study was too small. Reim et al. demonstrated that an elevation of AFP (>10 mg/L) can be considered as an independent poor prognostic predictor of OS and RFS in patients with GC using PSM (propensity score matching) analysis (10). This is similar to our findings.

Few studies had explored the effect of different serum AFP levels on prognosis of AFP-positive GC. In our research, we divided serum AFP level in AFP-positive GC into three subgroups and found that, with the increase of serum AFP level, the prognosis of patients also decreased. The 5-year survival rates for patients with AFP-positive GC with AFP ≤ 20 ng/ml, 20 < AFP ≤ 300 ng/ml, 300 < AFP ≤ 1,000 ng/ml, and AFP > 1,000 ng/ml were 51.3%, 30.2%, and 0%, respectively. This is consistent with a large-scale retrospective analysis carried out by Lin et al. (5). They revealed that the 5-year survival rates for patients with GC with AFP ≤ 20 ng/ml, 20 < AFP ≤ 300 ng/ml, and AFP > 300 ng/ml were 45.8%, 17.8%, and 0%, respectively. It demonstrated that the increase of AFP level is related to the poor prognosis of patients. Special attention should be paid to elevated serum AFP levels in patients with GC for their poor prognosis.

The molecular or cellular mechanisms leading to aggressive clinical behavior and poor prognosis of AFP-positive GC are still unclear. In HCC, AFP was not only known to be a product of tumor but also contributes to tumor aggression as well as regulation of hepatocellular growth and tumorigenesis (29). Similarly, AFP was reported to have immunosuppressive functions and inhibit the production of cytokines, interferons, and tumor necrosis factor by natural killer cells and macrophages (16). C-Met overexpression was found in AFP-positive GC when compared with AFP-negative GC (30, 31). Hepatocyte growth factor (HGF), a ligand for c-Met receptor, is strongly associated with malignant invasive property and development of distant metastases (30, 31). In addition, the frequency of VEGF-C expression in the AFP-positive GC was described significantly higher than that in the AFP-negative GC (32). AFP was considered to upregulate the VEGF-C expression, which may lead to unfavorable prognosis (33). The microvessel density in the AFP-positive GC was also higher than that in the AFP-negative GC. Therefore, it was described that the poorer prognosis of AFP-positive GC may be related to increased frequency of microvessel density and augmented expression of c-Met/HGF and VEGF-C (30–32).

Limitations of this study should not be ignored. Our study only included a small cohort of AFP-positive GC, whether it can represent the whole AFP-positive GC remains to be evaluated. In addition, selection bias in our retrospective study should be considered. Our findings should be validated in other cancer centers. In conclusion, a high level of serum AFP in GC was associated with a high incidence of lymph node metastasis, venous invasion, nerve invasion, liver metastasis, and a poor prognosis. Preoperative serum AFP levels could have predictive value for the development of liver metastasis in patients with GC. Therefore, serum AFP levels are an independent prognostic factor in GC.

## Data Availability Statement

The data are not publicly available due to privacy or ethical restrictions. The data that support the findings of this study are available on request from the corresponding author.

## Ethics Statement

The studies involving human participants were reviewed and approved by ethics committee of the Fujian Medical University Cancer Hospital, Fujian Cancer Hospital, Fuzhou, People’s Republic of China. The patients/participants provided their written informed consent to participate in this study.

## Author Contributions

The authors confirm contribution to the paper as follows: study conception and design: ZZ, XW, BC, and ZG; data collection: YZ, LP, JY, and JZ; analysis and interpretation of results: MS, ZZ, and XW; draft manuscript preparation: ZZ and BC. All authors contributed to the article and approved the submitted version.

## Funding

This study was supported by grants from the National Clinical Key Specialty Construction Program; Fujian Provincial Clinical Research Center for Cancer Radiotherapy and Immunotherapy (grant number: 2020Y2012); the Startup Fund for Scientific Research, Fujian Medical University (grant number: 2019QH1163); and Fujian Provincial Health Technology Project (grant numbers: 2019006 and 2021QNA040)

## Conflict of Interest

The authors declare that the research was conducted in the absence of any commercial or financial relationships that could be construed as a potential conflict of interest.

## Publisher’s Note

All claims expressed in this article are solely those of the authors and do not necessarily represent those of their affiliated organizations, or those of the publisher, the editors and the reviewers. Any product that may be evaluated in this article, or claim that may be made by its manufacturer, is not guaranteed or endorsed by the publisher.
